# Supplementary material for: Everyday norms have become more permissive over time and vary across cultures
Source: Commun Psychol. 2025 Oct 7;3:145. doi: 10.1038/s44271-025-00324-4 (PMC12504534; doi:10.1038/s44271-025-00324-4)
Supplement: Supplementary file 2 — Supplementary information [file 44271_2025_324_MOESM2_ESM.pdf]

Supplementary Information

Everyday norms have become more permissive over time and vary across cultures

Kimmo Eriksson et al.

Table of contents

Supplementary Table 1. Data collection sites and their sample characteristics.
Supplementary Table 2. Wording of the Ind-Bind Scale.
Supplementary Table 3. Wording of the version of the Moral Foundations Questionnaire used in the study.
Supplementary Table 4. Scores on the Ind-Bind scale in each society
Supplementary Table 5. Factor loadings etc. for the Ind-Bind scale.
Supplementary Table 6. Multilevel (cross-level vs. strong) invariance tests for the Ind-Bind scale.
Supplementary Table 7. Results from the preregistered analyses of the preregistered hypotheses H1-H5 on the preregistered sample.

Supplementary Table 8. Concerns about each situated behavior.
Supplementary Table 9. Full results for the Hypothesis on Societal Variation.
Supplementary Table 10. Full results for the Hypothesis on Change.

Supplementary Figure 1. A world map of societal variation in individualistic morality.
Supplementary Figure 2. Everyday norms across the globe with labels for rows and columns.
Supplementary Figure 3. Results on (A) societal variation and (B) change when including random slopes.
Supplementary Figure 4. Results on societal variation controlling for GDP per capita and the full set of demographic variables.
Supplementary Figure 5. Results on societal variation controlling for non-independence of countries.
Supplementary Figure 6. Results on societal variation with or without including the perceived commonness of situated behaviors.

Supplementary Table 1. Data collection sites and their sample characteristics.

Site	N	Age [SD]	Men	Women	Other	Missing gender	Students	Non-students	Missing student status
Algeria Oran	55	24.2 [8.3]	14.5%	85.5%	0.0%	0.0%	0.0%	0.0%	100.0%
Argentina Bahía Blanca	267	26.7 [8.4]	25.1%	65.5%	0.7%	8.6%	97.4%	2.6%	0.0%
Armenia Yerevan	466	19.7 [3.8]	24.0%	69.7%	4.1%	2.1%	100.0%	0.0%	0.0%
Australia National	564	43.1 [17.7]	30.7%	63.5%	0.2%	5.7%	0.0%	100.0%	0.0%
Azerbaijan Baku	290	31.3 [13.0]	39.0%	47.9%	1.0%	12.1%	0.0%	0.0%	100.0%
Bangladesh Chuadanga	169	24.6 [3.3]	55.0%	32.5%	0.0%	12.4%	0.0%	0.0%	100.0%
Bangladesh Dhaka	265	25.2 [5.6]	41.5%	40.0%	1.9%	16.6%	100.0%	0.0%	0.0%
Benin Cotonou	122	22.9 [5.1]	54.1%	45.9%	0.0%	0.0%	100.0%	0.0%	0.0%
Benin Parakou	100	22.9 [2.1]	60.0%	40.0%	0.0%	0.0%	100.0%	0.0%	0.0%
Bolivia National	659	26.0 [11.2]	41.9%	46.0%	2.0%	10.2%	0.0%	100.0%	0.0%
Bosnia and Herzegovina Sarajevo	243	33.7 [12.1]	22.6%	67.1%	0.8%	9.5%	0.0%	0.0%	100.0%
Brazil Centre West	149	23.3 [6.8]	46.3%	44.3%	2.7%	6.7%	89.3%	4.0%	6.7%
Brazil North	95	30.8 [13.4]	28.4%	55.8%	1.1%	14.7%	34.7%	50.5%	14.7%
Brazil Northeast	202	29.3 [14.1]	51.0%	47.0%	0.0%	2.0%	61.9%	36.1%	2.0%
Brazil South	219	22.8 [7.5]	53.4%	41.6%	1.4%	3.7%	90.0%	6.4%	3.7%
Brazil Southeast	245	39.1 [16.1]	35.9%	56.7%	2.4%	4.9%	32.7%	62.4%	4.9%
Bulgaria Plovdiv	105	29.1 [10.1]	21.0%	60.0%	1.9%	17.1%	100.0%	0.0%	0.0%
Central African Republic Bangui	258	25.3 [4.5]	39.1%	55.4%	3.1%	2.3%	88.8%	3.9%	7.4%
Cameroon Yaoundé	283	28.4 [6.6]	38.9%	47.0%	1.1%	13.1%	41.0%	45.2%	13.8%
Canada Kingston	261	19.6 [1.6]	54.4%	41.8%	2.7%	1.1%	100.0%	0.0%	0.0%
Canada Toronto	193	20.1 [4.4]	13.5%	84.5%	2.1%	0.0%	100.0%	0.0%	0.0%
Caribbean Regional	38	36.8 [14.6]	18.4%	63.2%	5.3%	13.2%	7.9%	92.1%	0.0%
Chad N’Djamena	49	21.5 [4.9]	34.7%	65.3%	0.0%	0.0%	100.0%	0.0%	0.0%
Chile Santiago	71	20.5 [1.8]	59.2%	35.2%	0.0%	5.6%	0.0%	0.0%	100.0%
China Beijing	209	23.2 [2.6]	31.1%	67.0%	1.0%	1.0%	100.0%	0.0%	0.0%
Colombia Bogota	334	29.5 [15.2]	37.4%	54.2%	2.7%	5.7%	0.0%	0.0%	100.0%
Costa Rica National	97	39.5 [12.1]	33.0%	43.3%	6.2%	17.5%	0.0%	0.0%	100.0%
Croatia Zagreb, Rijeka, Zadar, and Osijek	279	21.8 [3.9]	11.8%	83.2%	1.8%	3.2%	100.0%	0.0%	0.0%
Cuba Habana	279	21.1 [2.7]	36.9%	57.3%	1.1%	4.7%	100.0%	0.0%	0.0%
Czech Republic Brno	223	25.8 [10.0]	20.6%	72.2%	0.9%	6.3%	65.0%	28.7%	6.3%

Democratic Republic of the Congo Kinshasa area	281	21.9 [2.2]	31.3%	56.2%	0.7%	11.7%	0.0%	0.0%	100.0%
Denmark National	232	33.4 [15.4]	26.7%	62.1%	1.3%	9.9%	50.4%	40.1%	9.5%
El Salvador San Salvador	487	27.5 [10.0]	38.6%	55.9%	0.4%	5.1%	0.0%	0.0%	100.0%
Estonia Tallinn	252	33.1 [12.2]	34.1%	57.1%	0.4%	8.3%	0.0%	0.0%	100.0%
Ethiopia Several sites, mainly Addis Abeba	57	28.9 [6.4]	70.2%	24.6%	0.0%	5.3%	61.4%	38.6%	0.0%
Finland Helsinki area	343	28.1 [9.5]	17.2%	70.6%	7.9%	4.4%	100.0%	0.0%	0.0%
France Paris	80	37.2 [15.9]	15.0%	73.8%	6.2%	5.0%	0.0%	100.0%	0.0%
France National	153	46.9 [13.0]	49.7%	49.0%	0.7%	0.7%	0.0%	100.0%	0.0%
Georgia National	239	43.8 [17.4]	13.4%	61.9%	0.8%	23.8%	22.6%	53.6%	23.8%
Germany Cologne	221	28.1 [8.6]	35.3%	64.7%	0.0%	0.0%	0.0%	0.0%	100.0%
Gibraltar	83	42.4 [14.0]	43.4%	45.8%	6.0%	4.8%	26.5%	68.7%	4.8%
Greece National	160	35.7 [10.3]	26.2%	65.6%	1.2%	6.9%	0.0%	100.0%	0.0%
Greece Patra	282	21.5 [4.7]	36.2%	57.8%	2.5%	3.5%	100.0%	0.0%	0.0%
Guatemala Guatemala City	294	21.8 [6.6]	45.9%	39.5%	0.7%	13.9%	77.2%	8.2%	14.6%
Honduras Tegucigalpa	267	31.4 [11.8]	33.0%	55.4%	0.7%	10.9%	47.2%	52.8%	0.0%
Hungary Budapest	386	32.4 [16.9]	36.8%	59.1%	2.8%	1.3%	82.9%	17.1%	0.0%
Iceland Reykjavik	277	30.7 [12.6]	20.9%	66.4%	2.9%	9.7%	100.0%	0.0%	0.0%
India Mumbai	112	24.3 [5.8]	25.9%	61.6%	0.0%	12.5%	45.5%	42.0%	12.5%
India National	170	31.1 [9.9]	72.9%	24.7%	1.2%	1.2%	34.7%	64.1%	1.2%
Iran Tehran	263	28.8 [11.8]	28.1%	65.0%	1.5%	5.3%	57.4%	37.6%	4.9%
Ireland Dublin	250	20.3 [3.8]	34.0%	51.2%	2.0%	12.8%	100.0%	0.0%	0.0%
Israel National	326	32.8 [13.3]	39.6%	60.1%	0.3%	0.0%	62.9%	35.9%	1.2%
Italy National	210	32.8 [9.9]	55.7%	41.9%	1.9%	0.5%	35.7%	64.3%	0.0%
Italy Turin	227	21.2 [5.7]	29.5%	65.6%	0.9%	4.0%	100.0%	0.0%	0.0%
Jamaica (Caribbean)	20	42.0 [10.6]	5.0%	75.0%	0.0%	20.0%	100.0%	0.0%	0.0%
Japan Kanagawa	222	19.0 [0.8]	57.2%	41.4%	0.5%	0.9%	100.0%	0.0%	0.0%
Japan Nagoya	194	20.5 [1.5]	38.7%	59.3%	1.0%	1.0%	100.0%	0.0%	0.0%
Kazakhstan Almaty	208	20.4 [3.2]	45.2%	51.4%	2.9%	0.5%	100.0%	0.0%	0.0%
Kosovo Prishtina	204	24.9 [8.9]	12.7%	64.7%	2.5%	20.1%	100.0%	0.0%	0.0%
Kuwait Salmiya	231	28.3 [12.3]	34.6%	46.3%	0.0%	19.0%	0.0%	0.0%	100.0%
Latvia Daugavpils	204	35.8 [10.2]	19.1%	66.7%	5.9%	8.3%	79.4%	20.6%	0.0%

Lithuania National	225	48.4 [14.5]	44.0%	53.3%	0.4%	2.2%	0.0%	100.0%	0.0%
Malaysia Kuala Lumpur	382	24.2 [7.8]	27.2%	49.7%	4.2%	18.8%	68.1%	31.9%	0.0%
Malta National	225	36.7 [16.4]	24.4%	50.7%	0.9%	24.0%	33.3%	43.1%	23.6%
Martinique (Caribbean)	161	25.1 [7.4]	12.4%	66.5%	6.2%	14.9%	75.8%	24.2%	0.0%
Mauritius Port Louis	57	27.3 [9.6]	28.1%	70.2%	0.0%	1.8%	0.0%	0.0%	100.0%
Mexico National	109	23.7 [6.6]	11.0%	78.9%	0.0%	10.1%	58.7%	13.8%	27.5%
Mexico Guanajuato	360	28.0 [11.1]	40.3%	49.4%	2.2%	8.1%	0.0%	0.0%	100.0%
Moldova Chisinau	328	20.3 [4.4]	15.5%	76.5%	3.0%	4.9%	100.0%	0.0%	0.0%
Mongolia Ulaanbaatar	16	43.9 [12.8]	12.5%	68.8%	0.0%	18.8%	0.0%	100.0%	0.0%
Morocco Casablanca	214	23.4 [6.1]	24.3%	68.2%	0.0%	7.5%	100.0%	0.0%	0.0%
Mozambique Maputo	227	24.7 [8.3]	38.3%	56.8%	2.6%	2.2%	0.0%	0.0%	100.0%
Namibia Windoek	18	25.9 [5.3]	55.6%	22.2%	0.0%	22.2%	0.0%	0.0%	100.0%
The Netherlands Amsterdam	154	22.1 [4.8]	44.2%	47.4%	1.3%	7.1%	100.0%	0.0%	0.0%
New Zealand Dunedin	205	20.7 [4.8]	30.2%	65.4%	2.9%	1.5%	100.0%	0.0%	0.0%
Nigeria Nsukka	319	22.8 [4.3]	26.6%	48.3%	2.5%	22.6%	100.0%	0.0%	0.0%
North Macedonia Skopje	175	24.5 [7.7]	10.3%	80.0%	1.1%	8.6%	0.0%	0.0%	100.0%
Pakistan Balochistan	119	23.1 [4.2]	76.5%	17.6%	1.7%	4.2%	0.0%	0.0%	100.0%
Pakistan Gilgit Baltistan	56	24.2 [6.1]	41.1%	48.2%	1.8%	8.9%	0.0%	0.0%	100.0%
Pakistan Khyber Pakhtunkhwa	200	20.8 [2.9]	19.5%	73.5%	1.5%	5.5%	0.0%	0.0%	100.0%
Pakistan Punjab	420	24.5 [8.8]	40.5%	51.0%	1.0%	7.6%	0.0%	0.0%	100.0%
Pakistan Sindh	201	25.0 [8.8]	33.3%	51.2%	2.5%	12.9%	0.0%	0.0%	100.0%
Peru Lima	417	23.9 [9.0]	24.5%	61.4%	1.4%	12.7%	0.0%	94.0%	6.0%
Philippines Manila	218	21.7 [2.3]	30.3%	61.5%	3.7%	4.6%	0.0%	100.0%	0.0%
Poland National	138	46.1 [17.0]	57.2%	39.9%	0.0%	2.9%	0.0%	100.0%	0.0%
Poland Warsaw	228	25.3 [7.2]	20.2%	75.4%	0.4%	3.9%	100.0%	0.0%	0.0%
Portugal National	124	37.1 [10.5]	50.0%	49.2%	0.0%	0.8%	9.7%	90.3%	0.0%
Romania Timisoara	245	23.6 [7.9]	33.1%	65.3%	1.2%	0.4%	100.0%	0.0%	0.0%
Russia National	533	34.5 [13.6]	40.0%	59.3%	0.8%	0.0%	53.8%	46.2%	0.0%
Rwanda Kigali	242	25.1 [5.7]	50.4%	21.1%	14.9 %	13.6%	66.5%	20.2%	13.2%
Saudi Arabia Riyadh	81	27.9 [10.7]	85.2%	12.3%	0.0%	2.5%	0.0%	0.0%	100.0%
Serbia National	282	23.8 [7.8]	20.2%	69.9%	0.7%	9.2%	42.2%	47.5%	10.3%

Serbia Novi Sad	371	19.9 [2.1]	34.2%	62.3%	1.6%	1.9%	100.0%	0.0%	0.0%
Singapore	202	21.2 [1.6]	26.2%	72.8%	1.0%	0.0%	100.0%	0.0%	0.0%
Slovakia Trnava	229	26.0 [8.3]	17.0%	71.2%	0.4%	11.4%	100.0%	0.0%	0.0%
Slovenia Ljubljana	250	46.9 [11.8]	26.0%	65.6%	0.0%	8.4%	6.4%	93.6%	0.0%
Somalia Hargeisa	191	22.0 [2.7]	16.2%	67.5%	0.5%	15.7%	0.0%	0.0%	100.0%
South Korea Seoul	423	20.7 [2.8]	40.2%	55.6%	2.4%	1.9%	100.0%	0.0%	0.0%
South Korea National	122	43.1 [10.3]	49.2%	49.2%	0.0%	1.6%	0.0%	100.0%	0.0%
Spain Barcelona	201	24.6 [9.4]	44.3%	55.2%	0.5%	0.0%	0.0%	0.0%	100.0%
Sri Lanka Colombo	103	23.3 [3.0]	27.2%	44.7%	1.9%	26.2%	100.0%	0.0%	0.0%
Sweden Linkoping	225	28.1 [7.1]	37.3%	58.7%	1.8%	2.2%	57.8%	40.0%	2.2%
Sweden Lund	51	24.0 [3.9]	31.4%	54.9%	5.9%	7.8%	100.0%	0.0%	0.0%
Sweden Stockholm	24	27.2 [6.9]	29.2%	66.7%	0.0%	4.2%	100.0%	0.0%	0.0%
Taiwan	235	40.1 [12.7]	50.6%	47.7%	0.0%	1.7%	0.0%	100.0%	0.0%
Trinidad and Tobago (Caribbean)	35	27.2 [11.3]	37.1%	42.9%	5.7%	14.3%	100.0%	0.0%	0.0%
Turkey Istanbul	39	37.6 [13.8]	30.8%	61.5%	2.6%	5.1%	30.8%	56.4%	12.8%
Turkey National	216	30.3 [8.3]	31.0%	68.1%	0.9%	0.0%	56.0%	44.0%	0.0%
USA Athens, GA	398	18.7 [1.1]	19.1%	78.1%	1.5%	1.3%	100.0%	0.0%	0.0%
USA Columbia, SC	232	19.2 [2.0]	15.9%	83.2%	0.4%	0.4%	100.0%	0.0%	0.0%
USA New York, NY	199	27.8 [6.4]	47.7%	49.2%	2.5%	0.5%	40.7%	58.8%	0.5%
Uruguay Montevideo	214	29.9 [14.5]	25.7%	60.3%	0.5%	13.6%	86.4%	9.8%	3.7%
Vietnam Hanoi	146	19.6 [2.4]	11.0%	82.2%	2.1%	4.8%	100.0%	0.0%	0.0%
UK Cardiff	208	19.7 [6.0]	8.7%	88.9%	1.4%	1.0%	100.0%	0.0%	0.0%
Yemen Al Bayda', Sana'a	208	29.3 [8.0]	47.6%	33.2%	2.4%	16.8%	100.0%	0.0%	0.0%
Zambia Lusaka	302	22.0 [3.6]	33.8%	52.6%	1.7%	11.9%	100.0%	0.0%	0.0%
Zimbabwe Harare	194	21.9 [1.9]	17.5%	57.2%	1.5%	23.7%	100.0%	0.0%	0.0%

Note. In cases where Nonstudents is 100%, the general population was targeted which may have included student participants. In cases where Missing student status is 100%, both students and the general population were invited as participants but the question about student status was missing in the survey.

Supplementary Table 2. Wording of the Ind-Bind scale.

	What do you think is the right thing to do in a situation when someone must either
care_loyalty	(A) cause pain to someone or (B) be disloyal to their kin?
care_authority	(A) cause pain to someone or (B) break with a strong societal tradition?
care_purity	(A) cause pain to someone or (B) violate a religious rule?
fair_loyalty	(A) treat others unfairly or (B) be disloyal to their kin?
fair_authority	(A) treat others unfairly or (B) break with a strong societal tradition?
fair_purity	(A) treat others unfairly or (B) violate a religious rule?
liberty_loyalty	(A) restrict others' freedom to do as they want or (B) be disloyal to their kin?
liberty_authority	(A) restrict others' freedom to do as they want or (B) break with a strong societal tradition?
liberty_purity	(A) restrict others' freedom to do as they want or (B) violate a religious rule?

Note. The order between items and the order between clauses within an item (i.e., which was A and which was B) were both counterbalanced.

Supplementary Table 3. Wording of the version of the Moral Foundations Questionnaire used in the study.

Item type	Item
Individualizing	Compassion for those who are suffering is the most crucial virtue
	When the government makes laws, the number one principle should be ensuring that everyone is treated fairly
	One of the worst things a person could do is hurt a defenseless animal
	Justice is the most important requirement for a society
	It can never be right to kill a human being
	think it's morally wrong that rich children inherit a lot of money while poor children inherit nothing
	I think everyone should be free to do as they choose, so long as they don't infringe upon the equal freedom of others
	People should be free to decide what group norms or traditions they themselves want to follow
Binding	I am proud of my country's history
	Respect for authority is something all children need to learn
	People should not do things that are disgusting, even if no one is harmed
	People should be loyal to their family members, even when they have done something wrong
	Men and women each have different roles to play in society
	I would call some acts wrong on the grounds that they are unnatural
	It is more important to be a team player than to express oneself
	If I were a soldier and disagreed with my commanding officer's orders, I would obey anyway because that is my duty
	Chastity is an important and valuable virtue

Note. The order between items was counterbalanced.

Supplementary Table 4. Scores on the Ind-Bind scale in each society.

Country	Preregistered data	All data	Attention check	% passed attention check
Algeria		-1.29		21.8
Argentina	0.80	0.98	0.71	85.4
Armenia	-1.21	-1.02	-1.18	65.9
Australia	-0.46	-0.28	-0.33	75.4
Azerbaijan	0.00	0.12	0.12	40.0
Bangladesh	-1.31	-1.28	-1.39	30.4
Benin	-1.77	-1.63		95.0
Bolivia	-0.53	-0.35	-0.36	54.9
Bosnia and Herzegovina	0.42	0.58	0.35	64.6
Brazil	0.10	0.25	0.09	57.1
Bulgaria	0.10	0.31	0.08	61.0
Cameroon		-1.24	-1.15	30.4
Canada	-0.25	-0.12	-0.25	81.5
Caribbean	0.11	-0.34	-0.50	74.5
Central African Republic		-1.31		0.8
Chad		-1.14		10.2
Chile	0.39	0.53	0.47	73.2
China	0.10	0.25	0.03	87.1
Colombia	0.32	0.49	0.39	76.9
Costa Rica	0.86	1.05	0.82	86.6
Croatia	0.90	1.11	0.81	87.1
Cuba	0.41	0.59	0.44	71.0
Czechia	1.04	1.24	0.99	88.3
DRCongo	-1.43	-1.42	-1.42	15.7
Denmark	0.73	0.76	0.60	83.2
El Salvador	-0.33	-0.11	-0.20	59.3
Estonia	0.28	0.43	0.28	84.9
Ethiopia	-1.89	-1.76		31.6
Finland	1.26	1.44	1.17	93.3
France	0.62	0.77	0.52	89.3
Georgia		-0.06	0.08	47.7
Germany	1.00	1.19	0.94	92.8
Gibraltar	0.24	0.33	0.24	78.3
Greece	0.36	0.56	0.30	35.1
Guatemala	-0.14	-0.05	-0.14	77.9
Honduras	-0.26	-0.04	0.07	51.3
Hungary	0.23	0.40	0.23	60.9
Iceland	0.78	0.97	0.79	85.9
India	-0.41	-0.18	-0.23	64.5
Iran	-0.03	0.10	0.02	79.5
Ireland	0.90	1.05	0.86	89.2
Israel	0.04	0.23	0.03	93.9
Italy	0.96	1.16	0.96	84.2
Japan	0.50	0.69	0.43	84.6
Kazakhstan	0.38	0.25	0.11	70.7
Kosovo		-0.26	-0.03	49.0
Kuwait		-0.24	-0.30	64.9
Latvia	-0.64	-0.46	-0.45	55.9
Lithuania	-0.28	-0.10	-0.08	65.3

Country	Preregistered data	All data	Attention check	% passed attention check
Malaysia	0.02	0.08	0.02	66.2
Malta	0.55	0.68	0.52	71.1
Martinique	-0.55	-0.41	-0.70	58.4
Mauritius	-0.36	-0.27		59.6
Mexico	0.46	0.55	0.47	75.5
Moldova	0.02	0.18	0.20	46.0
Mongolia		-0.81		6.2
Morocco	-1.62	-1.43	-1.49	33.2
Mozambique	-1.10	-0.92	-0.81	10.1
Namibia		-0.46		33.3
Netherlands		0.78	0.48	90.3
New Zealand	0.38	0.59	0.41	85.9
Nigeria	-0.64	-0.40	-0.48	49.2
North Macedonia		0.12	0.17	64.6
Pakistan	-1.63	-1.45	-1.68	22.8
Peru	0.54	-0.35		47.5
Philippines	0.24	0.45	0.16	87.6
Poland	-0.29	-0.10	-0.15	82.2
Portugal		0.60	0.50	83.9
Romania	0.52	0.69	0.52	89.8
Russia		-0.49	-0.59	71.1
Rwanda	-0.92	-0.76	-0.50	8.3
Saudi Arabia		-1.88	-2.09	72.8
Serbia	-0.14	0.02	-0.04	70.1
Singapore	-0.10	0.09	-0.17	96.5
Slovakia	0.69	0.88	0.73	79.9
Slovenia	0.98	1.14	0.97	73.6
Somalia		-1.47		8.9
South Korea		0.71	0.54	82.9
Spain	0.75	0.94	0.71	89.1
Sri Lanka	0.31	0.25	0.30	48.5
Sweden	1.29	1.47	1.21	95.3
Taiwan		-0.43	-0.64	84.7
Turkey		0.27	-0.01	83.5
United Kingdom	0.47	0.67	0.44	91.3
United States	-0.09	0.10	-0.15	91.2
Uruguay	0.75	0.97	0.76	77.1
Vietnam		-0.75	-0.82	43.2
Yemen	-1.80	-1.54	-1.79	36.5
Zambia	-0.88	-0.79	-1.08	31.5
Zimbabwe	-0.74	-0.62	-0.86	45.4

Note. Ind-Bind factor scores, weighted by the factor loading of each item. The proportion of participants that passed the attention check varies immensely across societies. As there is considerable variation even among societies that used the same survey language, translation issues are unlikely to be the cause.

Supplementary Table 5. Factor loadings etc. for the Ind-Bind scale.

	Individual level				Society level			
Parameter	Est.	SE	p	Std.	Est.	SE	p	Std.
**Factor loadings**								
care_loyalty	0.302	0.008	<.001	0.296	0.302	0.008	<.001	0.663
care_authority	0.456	0.009	<.001	0.470	0.456	0.009	<.001	0.940
care_purity	0.616	0.010	<.001	0.610	0.616	0.010	<.001	1.000
fair_loyalty	0.227	0.009	<.001	0.219	0.227	0.009	<.001	0.533
fair_authority	0.460	0.009	<.001	0.458	0.460	0.009	<.001	0.904
fair_purity	0.613	0.011	<.001	0.581	0.613	0.011	<.001	0.973
liberty_loyalty	0.245	0.008	<.001	0.227	0.245	0.008	<.001	0.589
liberty_authority	0.474	0.010	<.001	0.437	0.474	0.010	<.001	0.911
liberty_purity	0.571	0.011	<.001	0.500	0.571	0.011	<.001	0.973
**Residual covariance**								
care_authority with care_purity	0.046	0.006	<.001	0.067				
care_loyalty with care_authority	0.063	0.005	<.001	0.076				
care_loyalty with care_purity	0.040	0.006	<.001	0.051				
fair_loyalty with fair_authority	0.060	0.006	<.001	0.067	0.016	0.003	<.001	0.297
fair_loyalty with fair_purity	0.044	0.006	<.001	0.050	0.018	0.004	<.001	0.507
fair_authority with fair_purity	0.027	0.006	<.001	0.035	0.011	0.002	<.001	0.493
liberty_loyalty with liberty_authority	0.132	0.006	<.001	0.128	0.014	0.003	<.001	0.275
liberty_loyalty with liberty_purity	0.137	0.006	<.001	0.132				
liberty_authority with liberty_purity	0.138	0.007	<.001	0.143				
care_authority with fair_authority	0.181	0.008	<.001	0.237	0.021	0.004	<.001	0.846
care_authority with liberty_authority	0.152	0.008	<.001	0.182	0.015	0.003	<.001	0.629
fair_authority with liberty_authority	0.150	0.008	<.001	0.173	0.018	0.004	<.001	0.573
care_purity with fair_purity	0.123	0.011	<.001	0.179				
care_purity with liberty_purity	0.132	0.011	<.001	0.167				
fair_purity with liberty_purity	0.174	0.011	<.001	0.205				
care_loyalty with fair_loyalty	0.286	0.007	<.001	0.290	0.069	0.011	<.001	0.806
care_loyalty with liberty_loyalty	0.272	0.007	<.001	0.265	0.071	0.011	<.001	0.891
fair_loyalty with liberty_loyalty	0.302	0.007	<.001	0.284	0.062	0.010	<.001	0.742
**Residual variance**								
care_loyalty	0.950	0.009	<.001	0.912	0.081	0.013	<.001	0.561
care_authority	0.732	0.009	<.001	0.779	0.019	0.004	<.001	0.116
care_purity	0.639	0.013	<.001	0.628	0.000	0.000		0.000
fair_loyalty	1.025	0.009	<.001	0.952	0.090	0.012	<.001	0.716
fair_authority	0.795	0.010	<.001	0.790	0.033	0.005	<.001	0.182
fair_purity	0.737	0.014	<.001	0.662	0.015	0.003	<.001	0.053
liberty_loyalty	1.105	0.010	<.001	0.949	0.078	0.012	<.001	0.653
liberty_authority	0.950	0.011	<.001	0.809	0.032	0.005	<.001	0.171
liberty_purity	0.978	0.014	<.001	0.750	0.013	0.003	<.001	0.053
**Factor variance**								
IM	1.000	0.000		1.000	0.693	0.107	<.001	1.000

Note. N = 25,422 in 90 societies. Factor variance at the individual level is set to 1. Factor loadings are restricted to be equal between the individual and the society level to reflect the across levels invariance. The table shows the best fitting partial invariance models. Residual variance at the society level was estimated freely for all items except for care_purity. Items are labeled as in Supplementary Table 2.

Supplementary Table 6. Multilevel (cross-level vs. strong) invariance tests for the Ind-Bind scale.

Sample	Model	df	Chi2	CFI	RMSEA	SRMR within	SRMR between
Preregistered	Two-level CFA	23	80.6	0.998	0.012	0.007	0.033
Preregistered	Cross-level invariance	35	197.6	0.995	0.016	0.008	0.057
Preregistered	Strong factorial invariance	53	3296.4	0.901	0.059	0.021	0.280
All data	Two-level CFA	23	93.4	0.998	0.011	0.006	0.026
All data	Cross-level invariance	35	225.5	0.995	0.015	0.007	0.061
All data	Strong factorial invariance	53	4943.6	0.884	0.060	0.018	0.286
Attention check	Two-level CFA	23	61.6	0.999	0.010	0.006	0.024
Attention check	Cross-level invariance	35	184.0	0.996	0.017	0.007	0.068
Attention check	Strong factorial invariance	53	3809.4	0.894	0.067	0.022	0.315

Note. The "Two-level CFA" specifies a single factor model with 9 indicators and covariances between the residuals from the same moral foundation. No constraints on factor loadings between levels were made. The "cross-level invariance" model constrains all factor loadings to be equal across the individual and country levels. The subsequent "strong factorial invariance" model sets the country level item residual variances to zero, which is equivalent to scalar invariance with multi-group CFA. As discussed by Jak & Jorgensen (2017; Relating measurement invariance, cross-level invariance, and multilevel reliability. *Frontiers in Psychology*, *8*, 1640), this two-step *cluster-bias* procedure is an omnibus test × a significant deterioration in fit when moving from the cross-level to the strong model tells us that either at least one loading or at least one intercept (or both) varies across clusters. The method therefore cannot isolate "metric" (loading) versus "scalar" (intercept) non-invariance in the classical single-level sense. Instead, it flags that strong invariance fails somewhere at Level 2.

Supplementary Table 7. Results from the preregistered analyses of the preregistered hypotheses H1-H5 on the preregistered sample.

	**H1**	**H2**	**H3**	**H4**	**H5**
Intercept	0.07 [−0.12, 0.25] 0.453	1.35 [1.13, 1.57] <0.001	−0.19 [−0.57, 0.18] 0.291	−0.16 [−0.49, 0.17] 0.305	−0.16 [−0.54, 0.22] 0.390
IM_c	0.33 [0.27, 0.39] <0.001	0.47 [0.40, 0.54] <0.001	0.28 [0.22, 0.34] <0.001		
IM_ic	0.14 [0.13, 0.15] <0.001	0.26 [0.24, 0.27] <0.001	0.11 [0.10, 0.12] <0.001		
Vulgar_b	−0.30 [−0.45, −0.16] <0.001	−0.27 [−0.46, −0.08] 0.009		−0.18 [−0.41, 0.04] 0.099	
Inconsiderate_b	−0.44 [−0.66, −0.23] <0.001			−0.20 [−0.52, 0.13] 0.202	
Inconsiderate_xb			−0.47 [−0.51, −0.44] <0.001		−0.52 [−0.58, −0.46] <0.001
Lacks sense_xb			−0.49 [−0.54, −0.44] <0.001		−0.54 [−0.62, −0.45] <0.001
Context: colleagues/classmates		−1.29 [−1.31, −1.28] <0.001			
Context: management/professors		−2.20 [−2.21, −2.18] <0.001			
Context: for a man		−0.29 [−0.30, −0.27] <0.001			
Context: for a woman		−0.07 [−0.08, −0.06] <0.001			
t				0.03 [0.01, 0.05] 0.004	0.04 [0.02, 0.06] <0.001
Age	−0.09 [−0.10, −0.09] <0.001	−0.12 [−0.13, −0.11] <0.001	−0.08 [−0.09, −0.08] <0.001	−0.10 [−0.11, −0.09] <0.001	−0.10 [−0.11, −0.09] <0.001
Woman	−0.05 [−0.07, −0.03] <0.001	−0.01 [−0.03, 0.01] 0.309	−0.07 [−0.09, −0.05] <0.001	−0.03 [−0.05, −0.01] 0.002	−0.03 [−0.05, −0.01] 0.002
IM_c × Vulgar_b	0.04 [0.04, 0.04] <0.001				
IM_ic × Vulgar_b	−0.01 [−0.01, −0.00] <0.001				
IM_c × Inconsiderate_b	−0.10 [−0.11, −0.10] <0.001				
IM_ic × Inconsiderate_b	−0.06 [−0.06, −0.05] <0.001				
IM_c × Inconsiderate_xb			−0.10 [−0.11, −0.10] <0.001		
IM_ic × Inconsiderate_xb			−0.05 [−0.05, −0.05] <0.001		
IM_c × Lacks sense_xb			−0.07 [−0.08, −0.07] <0.001		
IM_ic × Lacks sense_xb			−0.04 [−0.05, −0.04] <0.001		
t × Vulgar_b				−0.08 [−0.09, −0.08] <0.001	
t × Inconsiderate_b				−0.15 [−0.16, −0.15] <0.001	
t × Inconsiderate_xb					0.04 [0.03, 0.04] <0.001
Context: colleagues/classmates × Vulgar_b		−0.17 [−0.19, −0.16] <0.001			
Context: management/professors × Vulgar_b		−0.07 [−0.08, −0.06] <0.001			
Context: for a man × Vulgar_b		−0.26 [−0.27, −0.24] <0.001			
Context: for a woman × Vulgar_b		−0.18 [−0.19, −0.16] <0.001			
Context: colleagues/classmates × IM_c		−0.16 [−0.17, −0.14] <0.001			
Context: management/professors × IM_c		−0.17 [−0.18, −0.15] <0.001			

Context: for a man × IM_c		−0.00 [−0.02, 0.02] 0.785			
Context: for a woman × IM_c		0.11 [0.10, 0.13] <0.001			
Context: colleagues/classmates × IM_ic		−0.09 [−0.11, −0.07] <0.001			
Context: management/professors × IM_ic		−0.15 [−0.17, −0.14] <0.001			
Context: for a man × IM_ic		−0.04 [−0.06, −0.02] <0.001			
Context: for a woman × IM_ic		0.02 [0.00, 0.04] 0.020			
Context: alone × IM_c × Vulgar_b		0.08 [0.07, 0.09] <0.001			
Context: colleagues/classmates × IM_c × Vulgar_b		0.02 [0.01, 0.03] <0.001			
Context: management/professors × IM_c × Vulgar_b		−0.05 [−0.06, −0.04] <0.001			
Context: for a man × IM_c × Vulgar_b		0.04 [0.03, 0.05] <0.001			
Context: for a woman × IM_c × Vulgar_b		0.10 [0.09, 0.11] <0.001			
Context: alone × IM_ic × Vulgar_b		0.02 [0.01, 0.03] <0.001			
Context: colleagues/classmates × IM_ic × Vulgar_b		−0.03 [−0.04, −0.02] <0.001			
Context: management/professors × IM_ic × Vulgar_b		−0.04 [−0.05, −0.03] <0.001			
Context: for a man × IM_ic × Vulgar_b		−0.02 [−0.03, −0.00] 0.004			
Context: for a woman × IM_ic × Vulgar_b		0.03 [0.02, 0.04] <0.001			
SD society	0.19	0.22	0.19	0.24	0.24
SD individual	0.49	0.54	0.50	0.51	0.51
SD contextualized behavior	1.00		0.31	1.07	0.45
SD behavior	0.18	0.39	0.67	0.35	0.57
SD datapoint	1.13	1.13	1.12	1.10	1.10
$N_{individual}$	17,288	17,282	17,288	12,440	12,440
$N_{society}$	71	71	71	22	22
$N_{situated\ behavior}$	225	-	150	120	120
$N_{behavior}$	15	15	15	12	12
Num.Obs.	821,098	273,928	547,170	748,979	748,979
R2 Marg.	0.133	0.386	0.272	0.054	0.298
R2 Cond.	0.575	0.559	0.562	0.592	0.586

Note. IM_c and IM_ic refer to the factor scores of the Ind-Bind scale for the society and the individual, respectively. Vulgar_b and Inconsiderate_b are the concerns about vulgarity and inconsiderateness for behavior b, averaged across situations and centered at the mean across behaviors. Inconsiderate_xb and Lacks sense_xb are situation-specific measures of concerns about inconsiderateness and lacking sense, centered at the mean across situations for each behavior. The analysis of H2 includes dummy variables for each of the four social contexts (in front of colleagues/classmates, in front of management/professors, when a woman does it, when a man does it) with the asocial situation when "no one else is around" as the reference. Dummy variable t in the analysis of H4 and H5 is coded 1 for the new study, 0 for the old one (Gelfand et al., 2011). Age is measured in tens of years, centered on the global mean. Woman is dummy coded 1 for woman, 0 for man/other. SD is standard deviation. N is the number of cluster observations. The last three lines report the total number of observations and the marginal and conditional R-squared.

For H1, H3, H4 and H5, we obtain the same results in the preregistered analysis as we did in the merged models presented in the main text. H2, which was not included in the main text, was phrased: "Social context (who the bystanders are and who performs the behavior) has an effect on appropriateness ratings of everyday behaviors mainly for behaviors that elicit binding concerns, and this effect is larger in societies with less individualistic morality." The preregistered interpretation of the analysis was: "We expect that social context has an effect on appropriateness ratings of everyday

behaviors mainly for behaviors that elicit binding concerns (the coefficients for two-way interactions dummy x Vulgar_b are negative)." These coefficients are indeed negative, supporting the first part of the hypothesis. The interpretation continued: "and this effect is larger in societies and among individuals with less individualistic morality (the coefficients for three-way interactions dummy x Vulgar_b x IM_c are negative)." The signs of these coefficients are mixed, indicating a more complex relationship than expected. The social context data will be more thoroughly analyzed elsewhere.

Supplementary Table 8. Concerns about each situated behavior.

Vulgar	Inconsiderate	Lacks sense
1. kiss in a job interview (0.53)	1. talk in the library (0.69)	1. sing in a job interview (0.52)
2. flirt in a job interview (0.52)	2. argue at the movies (0.68)	2. cry in a job interview (0.51)
3. kiss at the workplace (0.47)	3. laugh out loud in the library (0.67)	3. read the newspaper at the movies (0.49)
4. curse/swear at the workplace (0.46)	4. talk at the movies (0.67)	4. argue in a job interview (0.48)
5. curse/swear at a party (0.45)	5. argue in the library (0.66)	5. listen to music on headphones in a job interview (0.48)
6. curse/swear in a restaurant (0.44)	6. sing in the library (0.64)	6. listen to music on headphones at the movies (0.47)
7. curse/swear at the movies (0.43)	7. use a mobile phone at the movies (0.62)	7. read the newspaper in a job interview (0.46)
8. curse/swear on a bus (0.43)	8. shout in anger in the library (0.61)	8. read the newspaper at a party (0.45)
9. curse/swear in the library (0.42)	9. argue at a funeral ceremony (0.61)	9. rest in a job interview (0.42)
10. curse/swear in a job interview (0.41)	10. laugh out loud at a funeral ceremony (0.58)	10. listen to music on headphones at a party (0.42)
11. kiss in the library (0.41)	11. sing at the movies (0.57)	11. use a mobile phone in a job interview (0.40)
12. kiss on a bus (0.41)	12. shout in anger at the movies (0.57)	12. laugh out loud in a job interview (0.40)
13. curse/swear in a public park (0.40)	13. listen to music on headphones at a funeral ceremony (0.56)	13. shout in anger in a job interview (0.37)
14. curse/swear on a city sidewalk (0.39)	14. read the newspaper at a funeral ceremony (0.55)	14. eat in a job interview (0.37)
15. flirt on a bus (0.38)	15. argue in a restaurant (0.54)	15. bargain at the movies (0.35)
16. flirt at the workplace (0.37)	16. shout in anger in a restaurant (0.53)	16. cry at the workplace (0.31)
17. kiss at the movies (0.37)	17. shout in anger at the workplace (0.52)	17. curse/swear in a job interview (0.29)

18. curse/swear at a funeral ceremony (0.37)
19. kiss on a city sidewalk (0.35)
20. kiss at a funeral ceremony (0.34)
21. kiss in a public park (0.34)
22. flirt at a funeral ceremony (0.33)
23. flirt on a city sidewalk (0.32)
24. kiss at a party (0.31)
25. kiss in a restaurant (0.30)
26. flirt in the library (0.27)
27. flirt at the movies (0.26)
28. flirt in a restaurant (0.26)
29. flirt in a public park (0.24)
30. shout in anger in a restaurant (0.23)
31. shout in anger in a job interview (0.22)
32. shout in anger on a city sidewalk (0.21)
33. shout in anger on a bus (0.20)
34. shout in anger in a public park (0.19)
35. argue on a bus (0.18)
36. shout in anger at a party (0.17)
37. bargain at a funeral ceremony (0.17)

18. argue on a bus (0.52)
19. shout in anger on a bus (0.51)
20. use a mobile phone at a funeral ceremony (0.51)
21. argue at a party (0.50)
22. bargain at a funeral ceremony (0.50)
23. eat in the library (0.48)
24. sing on a bus (0.48)
25. argue in a public park (0.47)
26. laugh out loud on a bus (0.46)
27. shout in anger at a funeral ceremony (0.45)
28. flirt at a funeral ceremony (0.45)
29. laugh out loud at the movies (0.45)
30. use a mobile phone in a job interview (0.45)
31. argue on a city sidewalk (0.44)
32. use a mobile phone in the library (0.44)
33. argue at the workplace (0.43)
34. shout in anger at a party (0.43)
35. shout in anger in a public park (0.43)
36. sing at the workplace (0.42)
37. shout in anger on a city sidewalk (0.42)

18. argue at the workplace (0.29)
19. shout in anger on a city sidewalk (0.28)
20. bargain on a bus (0.28)
21. bargain in a restaurant (0.28)
22. shout in anger at a party (0.27)
23. rest on a city sidewalk (0.27)
24. rest at the workplace (0.26)
25. shout in anger in a public park (0.26)
26. bargain in the library (0.26)
27. bargain in a job interview (0.26)
28. read the newspaper at a funeral ceremony (0.25)
29. cry on a city sidewalk (0.25)
30. argue in a public park (0.25)
31. argue on a city sidewalk (0.25)
32. shout in anger at the workplace (0.24)
33. bargain at a party (0.23)
34. sing at the workplace (0.23)
35. rest at the movies (0.23)
36. argue at a party (0.22)
37. read the newspaper at the workplace (0.22)

38. flirt at a party (0.17)	38. talk at a funeral ceremony (0.41)	38. laugh out loud at a funeral ceremony (0.22)
39. eat in a job interview (0.17)	39. listen to music on headphones in a job interview (0.39)	39. cry at a party (0.22)
40. laugh out loud at a funeral ceremony (0.17)	40. curse/swear at a funeral ceremony (0.39)	40. bargain in a public park (0.21)
41. shout in anger at a funeral ceremony (0.16)	41. laugh out loud in a restaurant (0.39)	40. shout in anger on a bus (0.21)
42. shout in anger in the library (0.16)	42. sing at a funeral ceremony (0.39)	42. bargain at the workplace (0.21)
43. listen to music on headphones at a funeral ceremony (0.16)	43. sing in a restaurant (0.38)	43. sing on a bus (0.20)
44. argue in a restaurant (0.16)	44. laugh out loud at the workplace (0.38)	44. rest in a restaurant (0.20)
45. shout in anger at the movies (0.16)	45. curse/swear in the library (0.37)	45. flirt in a job interview (0.20)
46. argue at a party (0.15)	46. rest at a funeral ceremony (0.35)	46. curse/swear at the workplace (0.20)
47. argue on a city sidewalk (0.15)	47. eat in a job interview (0.35)	47. sing in a restaurant (0.20)
48. argue at a funeral ceremony (0.15)	48. bargain in the library (0.35)	48. argue on a bus (0.20)
49. argue in a job interview (0.14)	49. shout in anger in a job interview (0.34)	50. argue in a restaurant (0.20)
50. laugh out loud in the library (0.14)	50. rest in a job interview (0.33)	50. cry in a restaurant (0.20)
51. sing at a funeral ceremony (0.13)	51. eat on a bus (0.33)	51. listen to music on headphones in a restaurant (0.20)
52. argue in a public park (0.12)	52. kiss at a funeral ceremony (0.33)	52. rest at a party (0.20)
53. eat at a funeral ceremony (0.12)	53. curse/swear in a public park (0.33)	53. use a mobile phone at the workplace (0.19)
54. shout in anger at the workplace (0.12)	54. eat at a funeral ceremony (0.33)	54. shout in anger at a funeral ceremony (0.19)
55. argue at the workplace (0.12)	55. bargain at the movies (0.31)	54. shout in anger at the movies (0.19)

56. argue in the library (0.11)	56. use a mobile phone in a restaurant (0.31)	56. kiss at a funeral ceremony (0.19)
57. use a mobile phone at a funeral ceremony (0.11)	57. curse/swear on a bus (0.31)	57. bargain at a funeral ceremony (0.19)
58. laugh out loud in a job interview (0.11)	58. curse/swear at the movies (0.30)	58. curse/swear in a restaurant (0.19)
59. bargain in a restaurant (0.11)	59. read the newspaper in a job interview (0.30)	59. cry in a public park (0.19)
60. laugh out loud in a restaurant (0.10)	60. curse/swear in a restaurant (0.30)	61. listen to music on headphones at the workplace (0.19)
61. laugh out loud at the workplace (0.10)	61. curse/swear on a city sidewalk (0.30)	61. sing at a funeral ceremony (0.19)
62. bargain in a job interview (0.09)	62. listen to music on headphones at the workplace (0.29)	61. talk in a job interview (0.19)
63. laugh out loud on a city sidewalk (0.09)	63. flirt in the library (0.28)	63. curse/swear on a city sidewalk (0.19)
64. talk at a funeral ceremony (0.09)	64. bargain at a party (0.28)	64. curse/swear on a bus (0.19)
65. sing at the movies (0.09)	65. bargain on a bus (0.28)	64. kiss in a job interview (0.19)
66. argue at the movies (0.09)	66. bargain in a job interview (0.28)	66. shout in anger in the library (0.19)
67. read the newspaper at a funeral ceremony (0.09)	67. rest at the workplace (0.28)	67. talk at a funeral ceremony (0.18)
68. bargain in the library (0.08)	68. laugh out loud in a job interview (0.28)	68. sing at the movies (0.18)
69. eat on a city sidewalk (0.08)	69. bargain on a city sidewalk (0.28)	69. eat in the library (0.18)
70. laugh out loud at the movies (0.08)	70. argue in a job interview (0.28)	70. cry on a bus (0.18)
71. sing in the library (0.08)	71. curse/swear at the workplace (0.27)	71. curse/swear at a party (0.18)
72. bargain at a party (0.08)	72. flirt at the workplace (0.27)	72. flirt at the workplace (0.18)
73. laugh out loud in a public park (0.07)	73. use a mobile phone at the workplace (0.27)	73. cry in the library (0.18)
74. rest in a job interview (0.07)	74. bargain in a restaurant (0.26)	74. sing in the library (0.18)

75. laugh out loud at a party (0.07)

76. listen to music on headphones in a job interview (0.07)

77. bargain in a public park (0.07)

78. eat in the library (0.07)

79. laugh out loud on a bus (0.07)

80. use a mobile phone in a job interview (0.07)

81. bargain on a bus (0.07)

82. bargain at the movies (0.07)

83. rest on a city sidewalk (0.07)

84. talk in the library (0.06)

85. read the newspaper in a job interview (0.06)

86. rest at a funeral ceremony (0.06)

87. talk at the workplace (0.06)

88. rest in a restaurant (0.06)

89. eat on a bus (0.06)

90. bargain on a city sidewalk (0.06)

91. sing in a job interview (0.06)

92. rest on a bus (0.06)

93. talk at the movies (0.06)

74. kiss at the workplace (0.26)

76. bargain at the workplace (0.25)

77. eat at the movies (0.24)

78. kiss in the library (0.24)

79. use a mobile phone on a city sidewalk (0.24)

80. laugh out loud in a public park (0.24)

81. read the newspaper at the workplace (0.23)

82. talk on a bus (0.23)

83. curse/swear in a job interview (0.23)

84. curse/swear at a party (0.23)

85. cry in the library (0.23)

86. sing in a job interview (0.23)

87. flirt at the movies (0.22)

88. kiss on a bus (0.22)

89. rest in a restaurant (0.22)

90. listen to music on headphones at a party (0.22)

91. talk at the workplace (0.21)

92. read the newspaper at a party (0.21)

93. use a mobile phone at a party (0.21)

75. use a mobile phone on a city sidewalk (0.18)

76. shout in anger in a restaurant (0.18)

77. sing on a city sidewalk (0.18)

78. curse/swear at the movies (0.17)

78. laugh out loud at the workplace (0.17)

80. curse/swear in a public park (0.17)

81. listen to music on headphones at a funeral ceremony (0.17)

82. read the newspaper in a restaurant (0.17)

83. argue in the library (0.16)

84. rest in the library (0.16)

85. use a mobile phone at the movies (0.16)

86. read the newspaper on a city sidewalk (0.16)

87. argue at a funeral ceremony (0.16)

88. bargain on a city sidewalk (0.16)

89. rest at a funeral ceremony (0.16)

90. use a mobile phone at a party (0.16)

91. curse/swear at a funeral ceremony (0.16)

92. curse/swear in the library (0.16)

93. use a mobile phone in the library (0.15)

94. use a mobile phone at the movies (0.05)
95. talk on a city sidewalk (0.05)
96. sing in a restaurant (0.05)
97. listen to music on headphones at the workplace (0.05)
98. talk in a job interview (0.05)
99. sing on a city sidewalk (0.05)
100. eat in a public park (0.05)
101. bargain at the workplace (0.05)
102. listen to music on headphones in a restaurant (0.05)
103. sing at a party (0.04)
104. use a mobile phone at the workplace (0.04)
105. eat at the workplace (0.04)
106. eat at the movies (0.04)
107. use a mobile phone at a party (0.04)
108. cry on a bus (0.04)
109. rest at the workplace (0.04)
110. cry on a city sidewalk (0.04)
111. rest in a public park (0.04)
112. sing at the workplace (0.04)
113. listen to music on headphones at a party (0.04)
114. rest at a party (0.03)

94. kiss on a city sidewalk (0.21)
95. kiss in a job interview (0.21)
96. sing in a public park (0.20)
97. cry in a restaurant (0.20)
98. talk in a job interview (0.20)
99. eat at the workplace (0.20)
100. flirt in a job interview (0.20)
102. bargain in a public park (0.20)
102. rest in the library (0.20)
103. read the newspaper at the movies (0.20)
104. laugh out loud on a city sidewalk (0.19)
105. cry in a job interview (0.19)
106. cry at the workplace (0.18)
106. sing on a city sidewalk (0.18)
108. flirt on a bus (0.18)
109. kiss in a public park (0.18)
110. kiss at the movies (0.18)
111. laugh out loud at a party (0.18)
112. rest on a city sidewalk (0.18)
113. cry at a party (0.18)
114. rest at a party (0.17)

94. eat on a city sidewalk (0.15)
95. talk at the movies (0.15)
96. eat at a funeral ceremony (0.15)
97. laugh out loud in a restaurant (0.15)
98. argue at the movies (0.15)
99. laugh out loud on a city sidewalk (0.15)
100. sing in a public park (0.14)
101. flirt in the library (0.14)
102. flirt at a funeral ceremony (0.14)
103. listen to music on headphones in the library (0.14)
104. laugh out loud on a bus (0.14)
105. eat at the workplace (0.14)
106. flirt on a bus (0.14)
107. flirt on a city sidewalk (0.14)
108. laugh out loud in the library (0.13)
109. use a mobile phone at a funeral ceremony (0.13)
110. flirt at the movies (0.13)
111. use a mobile phone in a restaurant (0.13)
112. laugh out loud at the movies (0.13)
113. talk at the workplace (0.13)
114. eat at the movies (0.13)

115. read the newspaper at the movies (0.03)
116. talk at a party (0.03)
117. cry at a party (0.03)
118. sing on a bus (0.03)
119. cry in the library (0.03)
120. use a mobile phone in the library (0.03)
121. read the newspaper in a restaurant (0.03)
122. cry at the movies (0.03)
123. talk on a bus (0.03)
124. listen to music on headphones in the library (0.03)
125. read the newspaper at a party (0.03)
126. read the newspaper at the workplace (0.03)
127. use a mobile phone on a bus (0.03)
128. talk in a restaurant (0.03)
129. read the newspaper in the library (0.03)
130. rest at the movies (0.03)
131. eat at a party (0.03)
132. cry in a public park (0.03)
133. cry in a restaurant (0.03)
134. listen to music on headphones on a city

115. flirt in a public park (0.17)
116. flirt on a city sidewalk (0.17)
117. eat on a city sidewalk (0.17)
118. flirt in a restaurant (0.17)
119. listen to music on headphones in a restaurant (0.16)
120. kiss in a restaurant (0.16)
121. listen to music on headphones at the movies (0.16)
122. kiss at a party (0.16)
123. listen to music on headphones on a city sidewalk (0.16)
124. cry on a bus (0.15)
125. use a mobile phone on a bus (0.15)
126. talk on a city sidewalk (0.14)
127. listen to music on headphones in the library (0.13)
128. read the newspaper on a city sidewalk (0.13)
129. cry in a public park (0.13)
130. cry on a city sidewalk (0.13)
131. flirt at a party (0.12)
132. cry at the movies (0.12)
133. rest at the movies (0.12)
134. rest in a public park (0.11)

115. flirt in a restaurant (0.12)
116. cry at the movies (0.12)
117. kiss in the library (0.12)
118. flirt at a party (0.12)
119. rest in a public park (0.11)
120. use a mobile phone in a public park (0.11)
121. talk on a city sidewalk (0.11)
122. rest on a bus (0.11)
123. kiss at the workplace (0.11)
124. laugh out loud in a public park (0.11)
125. use a mobile phone on a bus (0.11)
126. talk on a bus (0.10)
127. eat on a bus (0.10)
128. kiss on a bus (0.10)
129. flirt in a public park (0.10)
130. talk in the library (0.10)
131. kiss on a city sidewalk (0.10)
132. listen to music on headphones on a city sidewalk (0.10)
133. kiss in a restaurant (0.09)
134. read the newspaper on a bus (0.09)

sidewalk (0.03)		
135. listen to music on headphones at the movies (0.03)	134. rest on a bus (0.11)	135. read the newspaper in the library (0.09)
136. sing in a public park (0.03)	136. use a mobile phone in a public park (0.10)	136. laugh out loud at a party (0.08)
137. use a mobile phone in a restaurant (0.03)	137. eat at a party (0.09)	138. kiss at a party (0.08)
138. cry in a job interview (0.02)	138. listen to music on headphones on a bus (0.09)	138. read the newspaper in a public park (0.08)
139. eat in a restaurant (0.02)	139. talk in a restaurant (0.09)	139. listen to music on headphones in a public park (0.08)
140. cry at the workplace (0.02)	140. talk at a party (0.09)	140. cry at a funeral ceremony (0.07)
141. listen to music on headphones in a public park (0.02)	141. eat in a public park (0.09)	141. kiss at the movies (0.07)
142. use a mobile phone on a city sidewalk (0.02)	142. read the newspaper in a restaurant (0.08)	142. eat in a public park (0.07)
143. talk in a public park (0.02)	143. sing at a party (0.08)	142. listen to music on headphones on a bus (0.07)
144. listen to music on headphones on a bus (0.02)	144. talk in a public park (0.08)	144. sing at a party (0.07)
145. read the newspaper in a public park (0.02)	145. cry at a funeral ceremony (0.07)	145. talk at a party (0.06)
146. read the newspaper on a city sidewalk (0.02)	146. listen to music on headphones in a public park (0.07)	146. eat at a party (0.06)
147. rest in the library (0.02)	147. read the newspaper in the library (0.06)	147. kiss in a public park (0.05)
148. use a mobile phone in a public park (0.01)	148. read the newspaper on a bus (0.05)	148. talk in a public park (0.05)
149. cry at a funeral ceremony (0.01)	149. eat in a restaurant (0.05)	149. eat in a restaurant (0.04)
150. read the newspaper on a bus (0.01)	150. read the newspaper in a public park (0.03)	150. talk in a restaurant (0.04)

Note. For any given situated behavior, a random selection of participants were asked what would be the main concern for someone who disapproves of it. In each column, situated behaviors are sorted by the proportion of participants who selected a specific concern (in parentheses).

Supplementary Table 9. Full results for the Hypothesis on Societal Variation.

	Preregistered	All data	Attention check
Intercept	-0.19 [-0.41, 0.02] 0.075	-0.25 [-0.45, -0.05] 0.019	-0.20 [-0.43, 0.03] 0.080
IM_c	0.28 [0.22, 0.34] <0.001	0.28 [0.24, 0.33] <0.001	0.28 [0.23, 0.34] <0.001
IM_{ic}	0.11 [0.10, 0.12] <0.001	0.10 [0.09, 0.11] <0.001	0.11 [0.10, 0.12] <0.001
$Vulgar_b$	-0.28 [-0.44, -0.12] 0.003	-0.29 [-0.44, -0.13] 0.002	-0.30 [-0.45, -0.14] 0.001
$Inconsiderate_b$	-0.38 [-0.58, -0.18] 0.001	-0.38 [-0.57, -0.18] 0.001	-0.35 [-0.54, -0.17] 0.001
$Inconsiderate_{xb}$	-0.48 [-0.51, -0.44] <0.001	-0.48 [-0.52, -0.45] <0.001	-0.46 [-0.50, -0.42] <0.001
Lacks $sense_{xb}$	-0.49 [-0.54, -0.44] <0.001	-0.49 [-0.54, -0.43] <0.001	-0.48 [-0.54, -0.43] <0.001
Age	-0.08 [-0.09, -0.08] <0.001	-0.08 [-0.09, -0.08] <0.001	-0.07 [-0.08, -0.06] <0.001
Woman	-0.07 [-0.09, -0.05] <0.001	-0.06 [-0.08, -0.05] <0.001	-0.05 [-0.06, -0.03] <0.001
$IM_c \times Vulgar_b$	0.03 [0.03, 0.04] <0.001	0.02 [0.02, 0.02] <0.001	0.06 [0.06, 0.07] <0.001
$IM_{ic} \times Vulgar_b$	-0.01 [-0.01, -0.01] <0.001	-0.01 [-0.01, -0.01] <0.001	0.00 [0.00, 0.00] 0.934
$IM_c \times Inconsiderate_b$	-0.09 [-0.10, -0.09] <0.001	-0.10 [-0.10, -0.09] <0.001	-0.05 [-0.05, -0.04] <0.001
$IM_{ic} \times Inconsiderate_b$	-0.05 [-0.06, -0.05] <0.001	-0.05 [-0.06, -0.05] <0.001	-0.04 [-0.04, -0.03] <0.001
$IM_c \times Inconsiderate_{xb}$	-0.10 [-0.11, -0.10] <0.001	-0.12 [-0.12, -0.11] <0.001	-0.06 [-0.06, -0.06] <0.001
$IM_{ic} \times Inconsiderate_{xb}$	-0.05 [-0.05, -0.05] <0.001	-0.06 [-0.06, -0.05] <0.001	-0.04 [-0.04, -0.03] <0.001
$IM_c \times$ Lacks $sense_{xb}$	-0.07 [-0.08, -0.07] <0.001	-0.08 [-0.08, -0.08] <0.001	-0.05 [-0.06, -0.05] <0.001
$IM_{ic} \times$ Lacks $sense_{xb}$	-0.04 [-0.05, -0.04] <0.001	-0.04 [-0.05, -0.04] <0.001	-0.03 [-0.03, -0.02] <0.001
SD individual	0.50	0.51	0.47
SD society	0.19	0.18	0.17
SD situated behavior	0.31	0.29	0.34
SD behavior	0.36	0.34	0.39
SD datapoint	1.11	1.13	1.07
$N_{individual}$	17,288	25,422	15,599
$N_{society}$	71	90	80
$N_{situated\ behavior}$	150	150	150
$N_{behavior}$	15	15	15
Num.Obs.	547,170	833,930	515,162
R2 Marg.	0.385	0.369	0.421
R2 Cond.	0.564	0.543	0.601

Note. IM_c and IM_{ic} refer to the factor scores of the Ind-Bind scale for the society and the individual, respectively. $Vulgar_b$ and $Inconsiderate_b$ are the concerns about vulgarity and inconsiderateness for behavior b, averaged across situations and centered at the mean across behaviors. $Inconsiderate_{xb}$ and Lacks $sense_{xb}$ are situation-specific measures of concerns about inconsiderateness and lacking sense, centered at the mean across situations for each behavior. Age is measured in tens of years, centered on the global mean. Woman is dummy coded 1 for woman, 0 for man/other. SD is standard deviation. N is the number of cluster observations. The last three lines report the total number of observations and the marginal and conditional R-squared.

Supplementary Table 10. Full results for the Hypothesis on Change.

	Preregistered	All data	Attention check
Intercept	-0.17 [-0.50, 0.17] 0.301	-0.16 [-0.49, 0.17] 0.309	-0.16 [-0.51, 0.18] 0.312
t	0.04 [0.02, 0.06] <0.001	0.04 [0.02, 0.06] <0.001	0.05 [0.03, 0.06] <0.001
$Vulgar_b$	-0.18 [-0.41, 0.05] 0.112	-0.18 [-0.42, 0.05] 0.113	-0.18 [-0.40, 0.04] 0.092
$Inconsiderate_b$	-0.20 [-0.53, 0.13] 0.197	-0.20 [-0.54, 0.13] 0.205	-0.17 [-0.46, 0.12] 0.222
$Inconsiderate_{xb}$	-0.58 [-0.68, -0.49] <0.001	-0.62 [-0.72, -0.52] <0.001	-0.49 [-0.58, -0.41] <0.001
Age	-0.07 [-0.08, -0.07] <0.001	-0.08 [-0.08, -0.07] <0.001	-0.07 [-0.08, -0.07] <0.001
Woman	-0.05 [-0.06, -0.03] <0.001	-0.05 [-0.07, -0.04] <0.001	-0.04 [-0.06, -0.03] <0.001
t × $Vulgar_b$	-0.09 [-0.09, -0.08] <0.001	-0.10 [-0.10, -0.10] <0.001	-0.10 [-0.11, -0.10] <0.001
t × $Inconsiderate_b$	-0.15 [-0.15, -0.14] <0.001	-0.16 [-0.17, -0.16] <0.001	-0.17 [-0.17, -0.16] <0.001
t × $Inconsiderate_{xb}$	0.04 [0.04, 0.05] <0.001	0.04 [0.04, 0.05] <0.001	0.00 [-0.01, 0.00] 0.034
SD individual	0.51	0.51	0.48
SD society	0.23	0.23	0.23
SD situated behavior	0.69	0.68	0.74
SD behavior	0.45	0.44	0.45
SD datapoint	1.10	1.10	1.08
$N_{individual}$	12,440	15,304	12,429
$N_{society}$	22	26	26
$N_{situated\ behavior}$	120	120	120
$N_{behavior}$	12	12	12
Num.Obs.	869,180	929,143	856,692
R2 Marg.	0.255	0.258	0.250
R2 Cond.	0.591	0.588	0.600

Note. Dummy variable t is coded 1 for the new study, 0 for the old one (Gelfand et al., 2011). $Vulgar_b$ and $Inconsiderate_b$ are the concerns about vulgarity and inconsiderateness for behavior b, averaged across situations and centered at the mean across behaviors. $Inconsiderate_{xb}$ is the situation-specific measure of concerns about inconsiderateness centered at the mean across situations for each behavior. Age is measured in tens of years, centered on the global mean. Woman is dummy coded 1 for woman, 0 for man/other. SD is standard deviation. N is the number of cluster observations. The last three lines report the total number of observations and the marginal and conditional R-squared.

Supplementary Figure 1. A world map of societal variation in individualistic morality. Brighter colors indicate more individualistic morality as measured by factor scores with MLSEM across 90 societies.

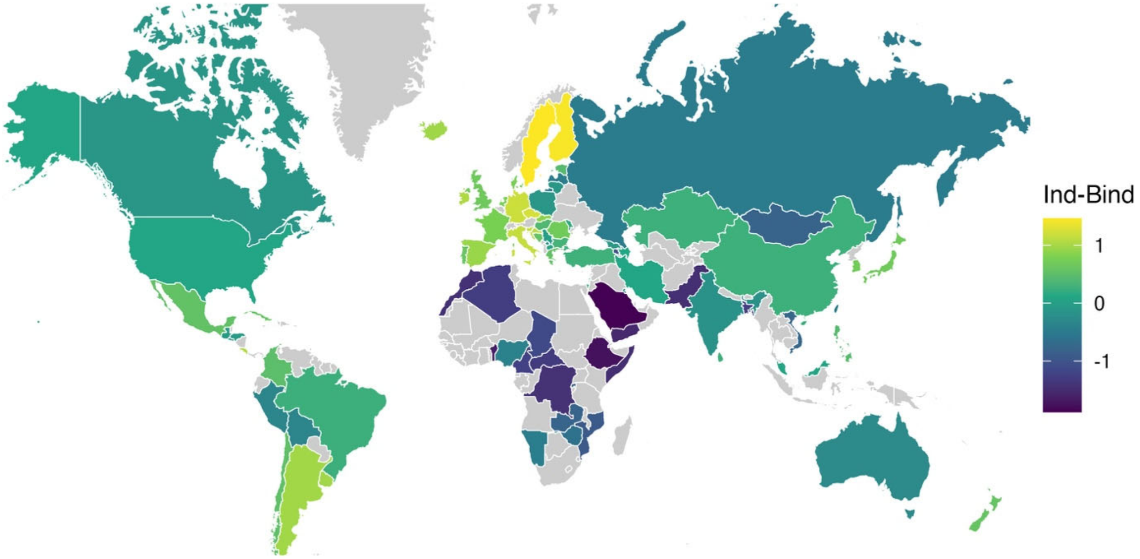

Supplementary Figure 2. Everyday norms across the globe with labels for rows and columns. (Three pages.) N = 54.3 appropriateness ratings on average, ranging from 1 to 217 across cells, coded -2.5 - extremely inappropriate (light color) to 2.5 - extremely appropriate (dark color).

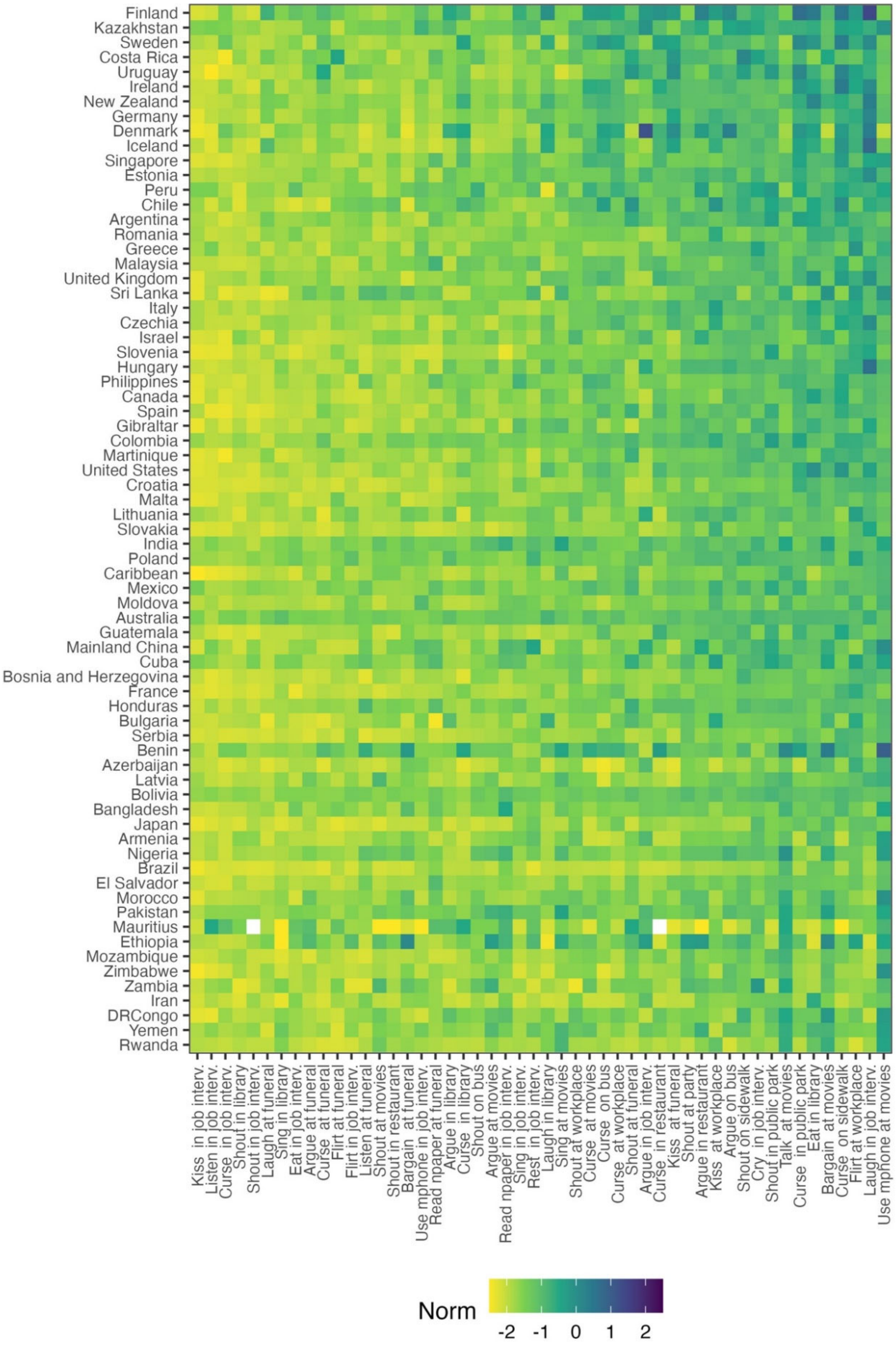

Supplementary Figure 2. (2nd page.)

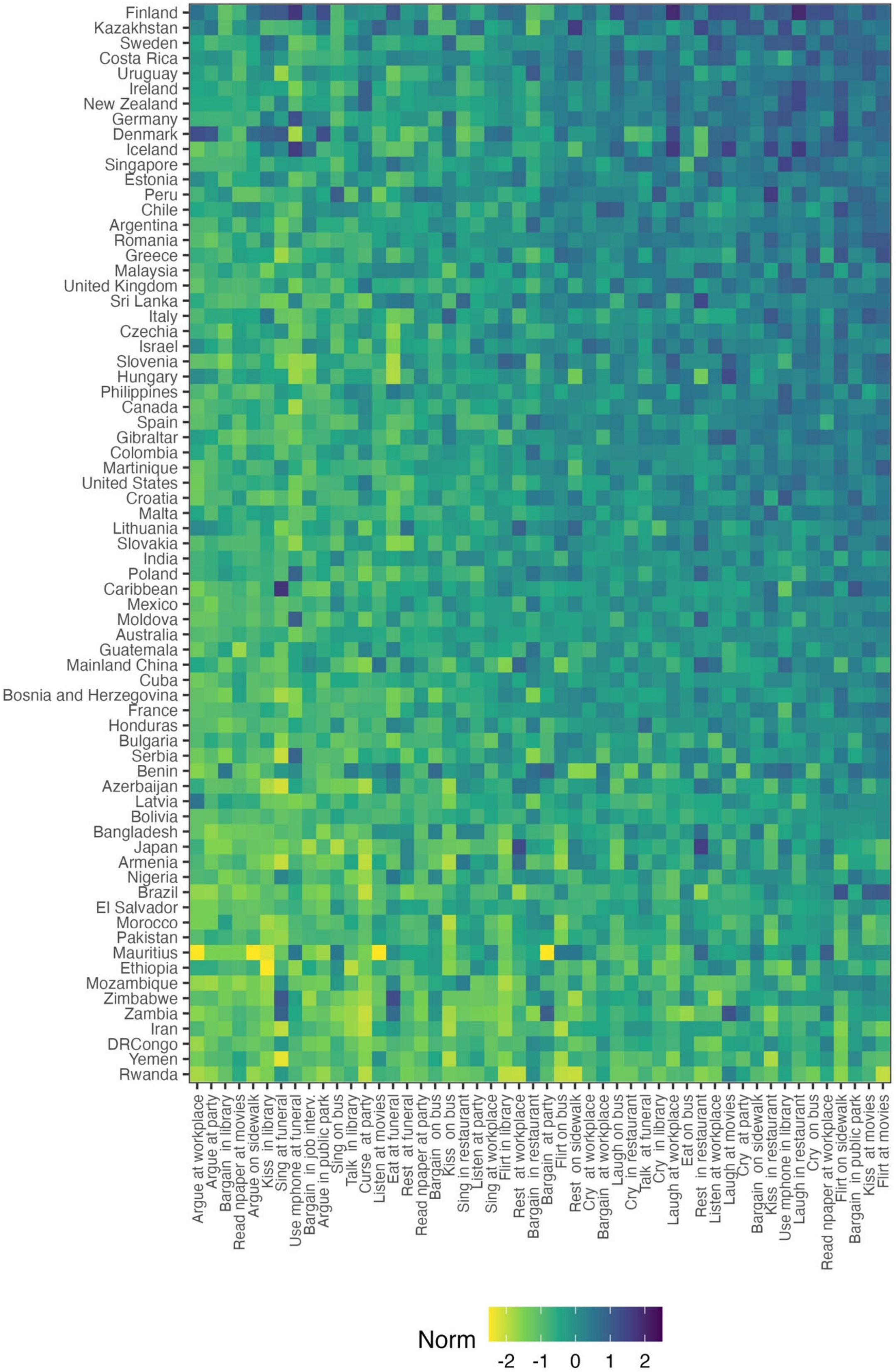

Supplementary Figure 2. (3rd page.)

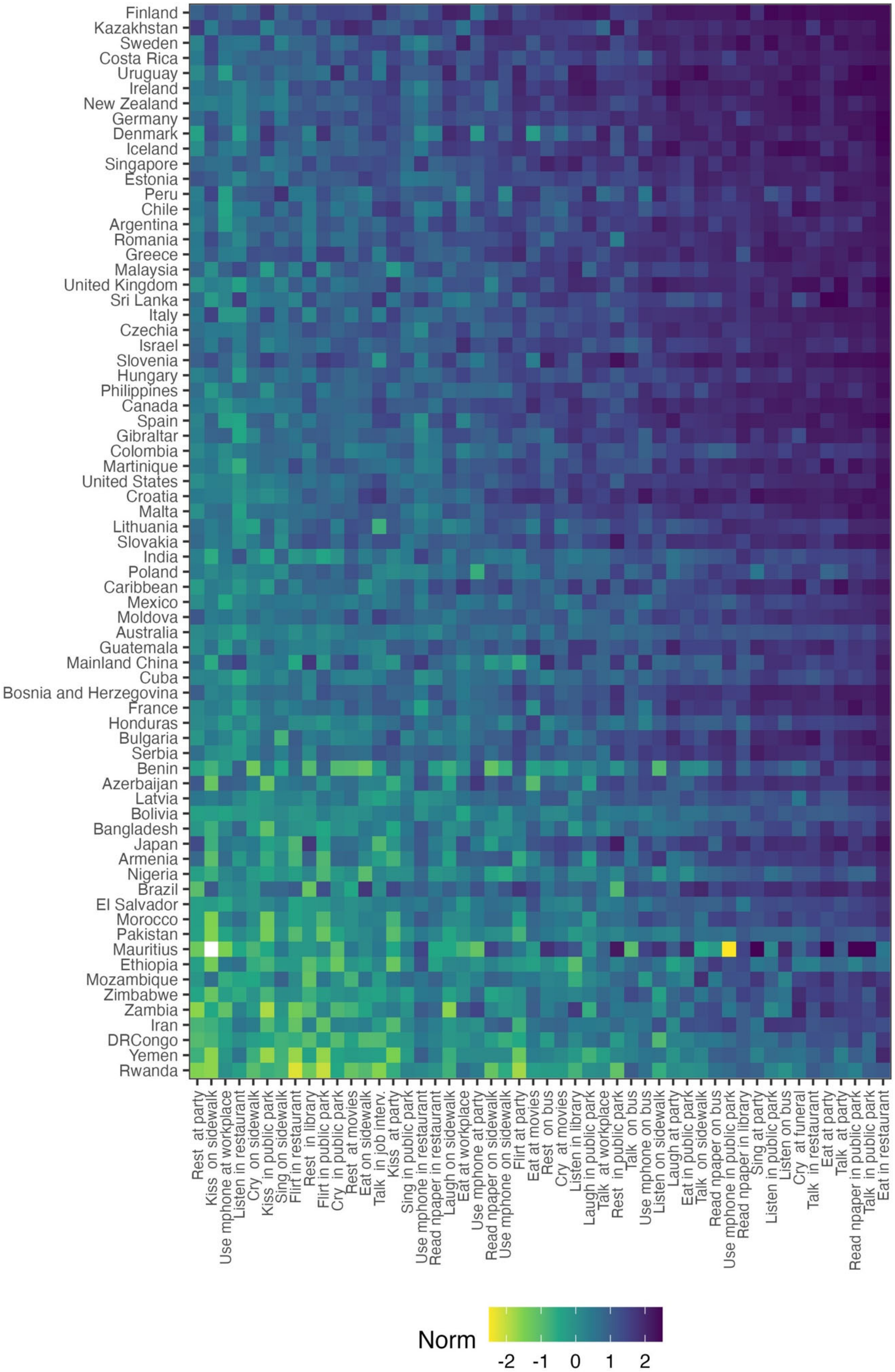

Supplementary Figure 3. Results on (A) societal variation and (B) change when including random slopes.

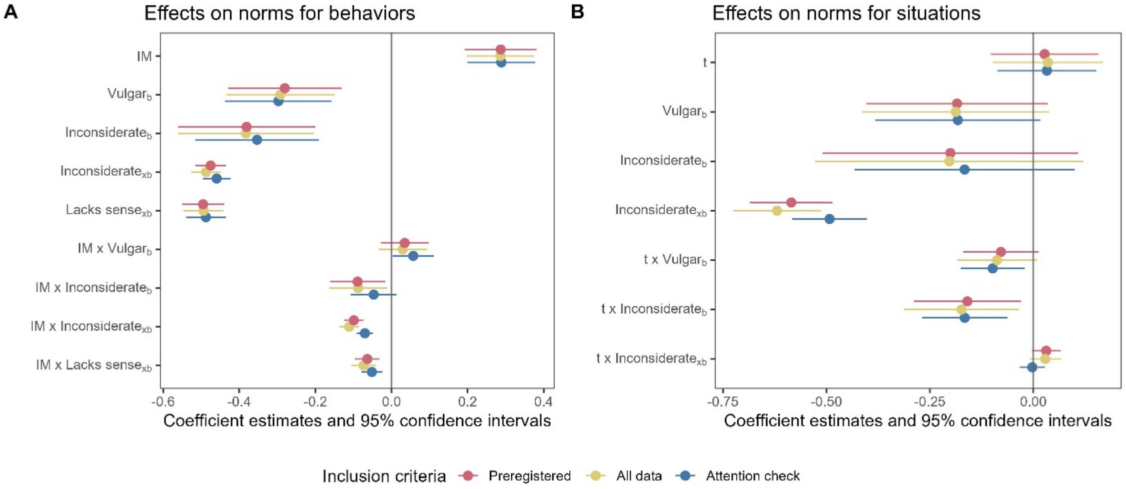

Note. The analysis extends the models of societal variation and change with a set of random slopes for the predictors involved in the country-level interactions. In panel A, the society-level effect of individualistic morality is varied by behavior-situation and by behavior (that is β_1 in Eq. 1 is replaced by $\beta_1+v_{1xb}+v_{2b}$), the behavior-specific effects of Vulgar and Inconsiderate are varied by society (β_6 and β_7 become β_6+v_{6c} and β_7+v_{7c}, respectively), and the situation-specific effects of Inconsiderate and Lacks sense are varied by society (β_8 and β_9 become β_8+v_{8c} and β_9+v_{9c}, respectively). To speed-up convergence we dropped random intercept for the individual level (u_{4ic}). The number of observations are 547,170 from 71 countries in the P*reregistered* sample (red), 833,930 from 90 countries in *All data* (yellow), and 515,162 from 80 countries in the *Attention check* (blue). In panel B, the effect of time is varied by behavior-situation and by behavior (that is β_1 in Eq. 1 is replaced by $\beta_1+v_{1xb}+v_{2b}$), the behavior-specific effects of Vulgar and Inconsiderate are varied by society (β_6 and β_7 become β_6+v_{6c} and β_7+v_{7c}, respectively), and the situation-specific effects of Inconsiderate is varied by society (β_8 become β_8+v_{8c}). The number of observations are 869,180 from 22 countries in the P*reregistered* sample (red), 929,143 from 26 countries in *All data* (yellow), and 856,692 from 26 countries in the *Attention check* (blue).

Supplementary Figure 4. Results on societal variation controlling for GDP per capita and the full set of demographic variables.

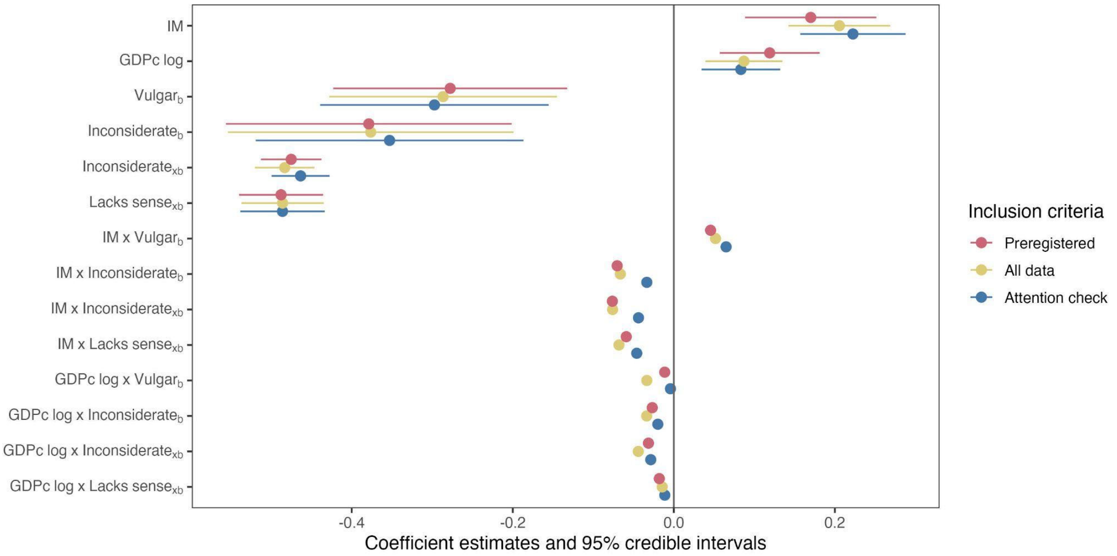

Note. GDPc log is log transformed GDP per capita adjusted for purchasing power parity. The data is not available for Cuba, Gibraltar, Kosovo, and Martinique. The number of observations are 524,465 from 69 countries in the P*reregistered* sample (red), 811,038 from 86 countries in *All data* (yellow), and 501,736 from 76 countries in the *Attention check* (blue).

Supplementary Figure 5. Results on societal variation controlling for non-independence of countries.

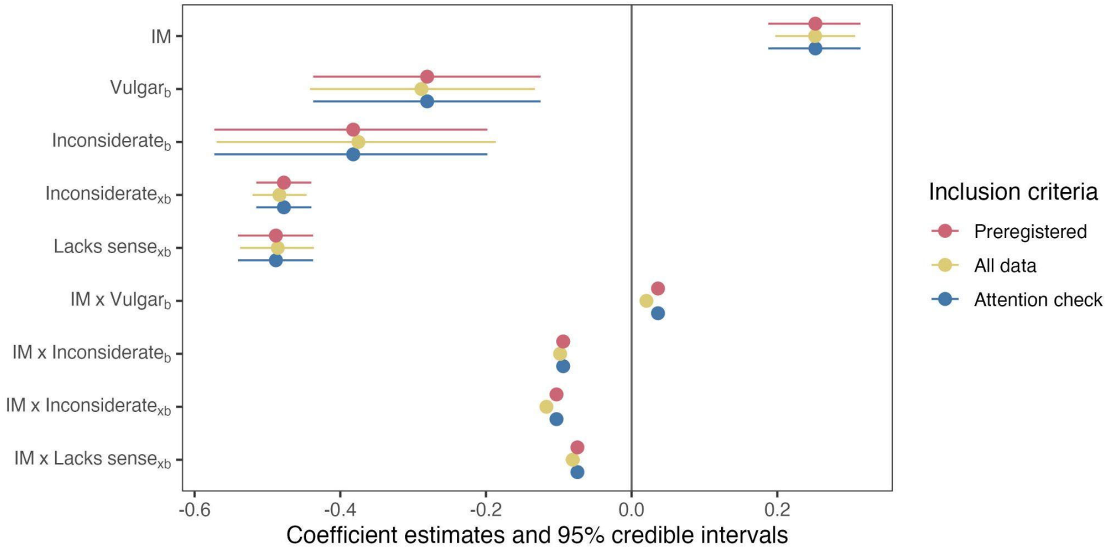

Note. This model does not include the individual level because it is very computing intensive. Data are aggregated by society, weighted by the number of ratings over which appropriateness ratings are estimated. To control for non-independence of societies, we included society-level random intercepts allowed to covary according to geographic and linguistic proximity. Geographic proximity was modelled using Gaussian processes on country centroids (longitude, latitude), and linguistic proximity was derived from a normalised linguistic distance matrix (no data for Kosovo; for the Caribbean society, proximity values for Trinidad and Tobago were used). The number of observations are 10,646 aggregated ratings for 150 situated behaviors from 71 countries in the P*reregistered* sample (red), 13,318 from 89 countries in *All data* (yellow), and 11,820 from 79 countries in the *Attention check* (blue).

Supplementary Figure 6. Results on societal variation with or without including the perceived commonness of situated behaviors.

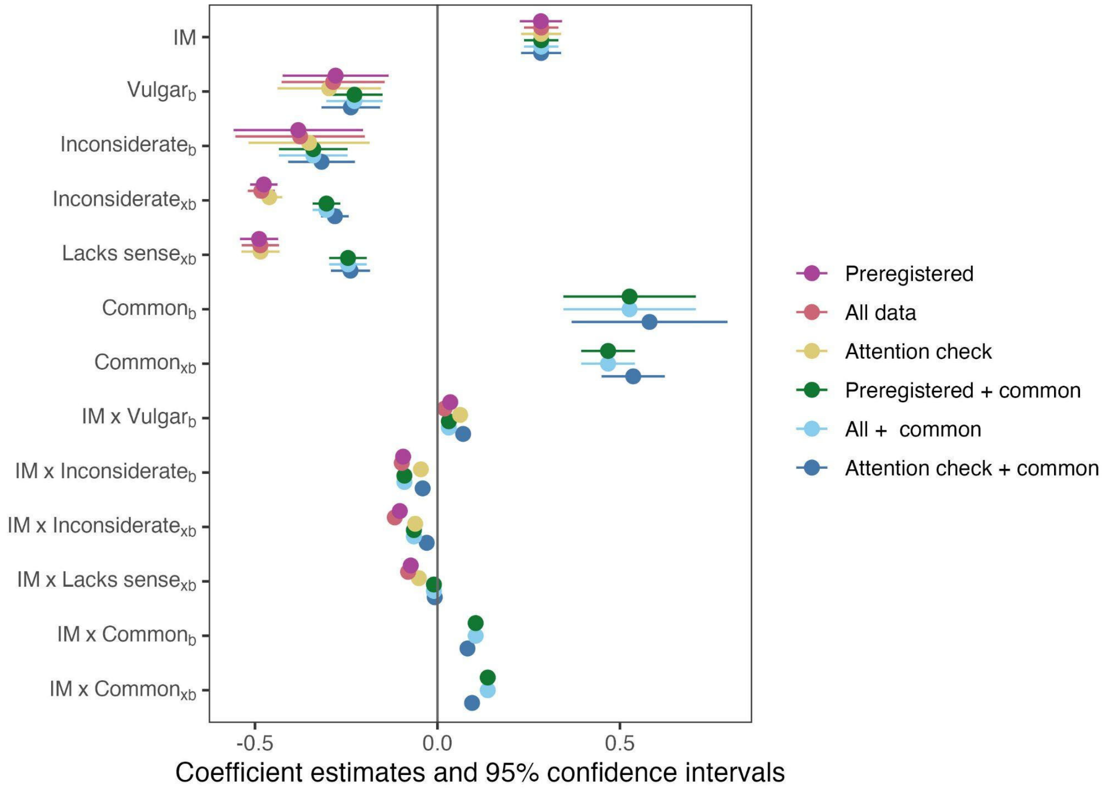

Note. Common_b and Common_{xb} indicate behavior-specific and situation-specific commonness. The sample size remains unchanged.
