# Supplementary material for: Everyday norms have become more permissive over time and vary across cultures
Source: Commun Psychol. 2025 Oct 7;3:145. doi: 10.1038/s44271-025-00324-4 (PMC12504534; doi:10.1038/s44271-025-00324-4)
Supplement: Supplementary file 3 — Reporting summary [file 44271_2025_324_MOESM3_ESM.pdf]

Corresponding author(s): Kimmo Eriksson

Last updated by author(s): Aug 13, 2025



# Reporting Summary

Nature Portfolio wishes to improve the reproducibility of the work that we publish. This form provides structure for consistency and transparency in reporting. For further information on Nature Portfolio policies, see our Editorial Policies and the Editorial Policy Checklist.

## Statistics

For all statistical analyses, confirm that the following items are present in the figure legend, table legend, main text, or Methods section.

| n/a | Confirmed | |
|---|---|---|
| ☐ | ☒ | The exact sample size (*n*) for each experimental group/condition, given as a discrete number and unit of measurement |
| ☐ | ☒ | A statement on whether measurements were taken from distinct samples or whether the same sample was measured repeatedly |
| ☐ | ☒ | The statistical test(s) used AND whether they are one- or two-sided<br>*Only common tests should be described solely by name; describe more complex techniques in the Methods section.* |
| ☐ | ☒ | A description of all covariates tested |
| ☐ | ☒ | A description of any assumptions or corrections, such as tests of normality and adjustment for multiple comparisons |
| ☐ | ☒ | A full description of the statistical parameters including central tendency (e.g. means) or other basic estimates (e.g. regression coefficient) AND variation (e.g. standard deviation) or associated estimates of uncertainty (e.g. confidence intervals) |
| ☐ | ☒ | For null hypothesis testing, the test statistic (e.g. *F*, *t*, *r*) with confidence intervals, effect sizes, degrees of freedom and *P* value noted<br>*Give P values as exact values whenever suitable.* |
| ☐ | ☒ | For Bayesian analysis, information on the choice of priors and Markov chain Monte Carlo settings |
| ☐ | ☒ | For hierarchical and complex designs, identification of the appropriate level for tests and full reporting of outcomes |
| ☐ | ☒ | Estimates of effect sizes (e.g. Cohen's *d*, Pearson's *r*), indicating how they were calculated |

*Our web collection on statistics for biologists contains articles on many of the points above.*

## Software and code

Policy information about availability of computer code

| | |
|---|---|
| Data collection | Qualtrics survey builder was used for data collection. |
| Data analysis | We used R version 4.3.1. The R syntax for all analyses are available at OSF (https://osf.io/sh4rb/, DOI: 10.17605/OSF.IO/SH4RB). |

For manuscripts utilizing custom algorithms or software that are central to the research but not yet described in published literature, software must be made available to editors and reviewers. We strongly encourage code deposition in a community repository (e.g. GitHub). See the Nature Portfolio guidelines for submitting code & software for further information.

## Data

Policy information about availability of data

All manuscripts must include a data availability statement. This statement should provide the following information, where applicable:

- Accession codes, unique identifiers, or web links for publicly available datasets
- A description of any restrictions on data availability
- For clinical datasets or third party data, please ensure that the statement adheres to our policy

All data and materials generated and/or analyzed in this study, including the raw data underlying the figures and tables, are available at OSF (https://osf.io/sh4rb/, DOI: 10.17605/OSF.IO/SH4RB).

## Research involving human participants, their data, or biological material

Policy information about studies with human participants or human data. See also policy information about sex, gender (identity/presentation), and sexual orientation and race, ethnicity and racism.

| | |
|---|---|
| Reporting on sex and gender | Participants self-reported gender: 33.4% men, 57.8% women, 1.8% other, 7.1% missing data. Gender was not part of the study design but the preregistered analyses included gender as a control; the observed effect of gender is reported in Supplementary Table 7. |
| Reporting on race, ethnicity, or other socially relevant groupings | No data on race or ethnicity was collected. Participants in this international study are grouped by their society (country or corresponding) as defined by where they took the survey (i.e., not self-reported). |
| Population characteristics | See below. |
| Recruitment | Researchers in each society were instructed to obtain a local sample of participants aged 18 and above, if possible both students and non-students. How to recruit participants and whether to compensate them was up to each site to decide. Participants were recruited via various methods (e.g., email, social media, survey organizations), and compensation varied by site (e.g., monetary, course credit, vouchers). |
| Ethics oversight | Ethics committees and institutional review boards approved the study protocol (or decided that the study was exempt from ethical approval) where required, including the Swedish Ethical Review Authority (Sweden), Macquarie University (Australia); Queen's University General Research Ethics Board (GREB) (Canada), Department of Psychology Board of Ethics, Faculty of Humanities and Social Sciences, University of Zagreb (Croatia); Université Protestante au Congo (Democratic Republic of Congo); Paris School of Economics (France); Research and Research Degrees Committee, University of Gibraltar (Gibraltar); Research Ethics Committee (REC) (Greece); United Psychological Research Ethics Committee (Hungary); Monk Prayogshala Institutional Review Board (India); Aoyama Gakuin University Research Ethics Committee (Japan); Ethics Committee of Graduate School of Informatics (Japan); Institutional Review Board Committee, American University of Kuwait (Kuwait); Sunway University Research Ethics Committee (Malaysia); Faculty (FEMA) Research Ethics Committee (Malta); University of Otago Ethics Committee (New Zealand); Ethics Subcommittee of the Macedonian Academy of Sciences and Arts (MASA) (North Macedonia); Ethical Review Board at SWPS University Faculty of Psychology in Warsaw (Poland); Scientific committee of the Center for Social Diagnosis (CDS) (Romania); Ethics Committee of the Department of Psychology, Faculty of Philosophy in Novi Sad. (Serbia); Department of Psychology, Faculty of Philosophy, University of Niš, Serbia (Serbia); Singapore Management University IRB (Singapore); Ethics Committee of the Center for Social and Psychological Sciences, Slovak Academy of Sciences (Slovakia); IRB at IESE Business School (Spain); Post Graduate Institute of Medicine, University of Colombo (Sri Lanka); Koç University (Turkey); New York University IRB (U.S.A); University of Georgia IRB (U.S.A); Institutional Review Board, University of South Carolina (U.S.A); Cardiff School of Psychology's Research Ethics Committee (UK); Comité de Ética en Investigación/Universidad Católica del Uruguay (Uruguay); Ethics Committee of the Dept of Education and Psychology, Forman Christian College (Pakistan); Saint George's University IRB (Grenada). |

Note that full information on the approval of the study protocol must also be provided in the manuscript.

# Field-specific reporting

Please select the one below that is the best fit for your research. If you are not sure, read the appropriate sections before making your selection.

☐ Life sciences ☒ Behavioural & social sciences ☐ Ecological, evolutionary & environmental sciences

For a reference copy of the document with all sections, see nature.com/documents/nr-reporting-summary-flat.pdf

# Behavioural & social sciences study design

All studies must disclose on these points even when the disclosure is negative.

| | |
|---|---|
| Study description | The study was designed to test the predictions of our theoretical model of everyday norms, according to which the strength of the norm against a given situated behavior in a given society is determined by a specific interaction between the society's level of individualizing morality and the concerns elicited by the situated behavior (assumed society-independent). The study is a within-subject experiment that manipulated situations and behaviors; specifically, we fully crossed 15 common behaviors with 10 familiar situations. For each situated behavior, we measured the strength of the norm against a certain situated behavior in a given society (operationalized as the average rating of the inappropriateness of that situated behavior among participants in that society) and the everyday concerns it elicits (operationalized as the frequencies at which participants in the global sample selected what concerns would be the main concern for someone who disapproves of the behavior in that situation). |
| Research sample | We achieved variation in individualizing morality by sampling 90 societies across the globe, including very liberal societies (e.g., Sweden) and very morally conservative societies (e.g., Saudi Arabia). Data were collected from convenience samples with a mix of students (55%) and non-students (23%; for the remaining 22% this data is missing). Supplementary Table 1 reports, for each society in the study, sample characteristics with respect to age, gender, and student/nonstudent status. We check that societal differences in values obtained in the World Values Survey are well-captured by our samples. |
| Sampling strategy | |

| | |
|---|---|
| Sampling strategy | We set a target sample size of at least 200 participants per society in 80 societies but with the understanding that sites with small populations to sample from might not reach the target sample size. |
| Data collection | The data was collected anonymously online using Qualtrics, with exception for the data from Mauritius and Benin where the same questions were asked face-to-face by an interviewer who recorded the responses in Qualtrics. |
| Timing | Data were collected between July 14, 2023 and May 31, 2024. |
| Data exclusions | Analyses of three samples are reported based on (1) Preregistered exclusions, (2) All data, no exclusions, and (3) exclusion of participants who failed attention check. |
| Non-participation | To take the survey, participants needed to give their informed consent, make a commitment to give their best answers (see below), and report an age of 18 or above. For either of these reasons, 1,870 potential participants who entered the survey were not allowed to take it. Another 4,913 dropped out of the survey before answering any questions about social norms and therefore did not provide any data for this paper. |
| Randomization | Behaviors and situations were manipulated within subjects. However, to limit participant fatigue, each participant only rated norms for a subset (< 60) of situated behaviors randomly drawn from the full set. In the preregistered analyses, this is handled by including random intercepts for individuals as well as for situated behaviors. |

# Reporting for specific materials, systems and methods

We require information from authors about some types of materials, experimental systems and methods used in many studies. Here, indicate whether each material, system or method listed is relevant to your study. If you are not sure if a list item applies to your research, read the appropriate section before selecting a response.

## Materials & experimental systems

| n/a | Involved in the study | |
|---|---|---|
| ☒ | ☐ | Antibodies |
| ☒ | ☐ | Eukaryotic cell lines |
| ☒ | ☐ | Palaeontology and archaeology |
| ☒ | ☐ | Animals and other organisms |
| ☒ | ☐ | Clinical data |
| ☒ | ☐ | Dual use research of concern |
| ☒ | ☐ | Plants |

## Methods

| n/a | Involved in the study | |
|---|---|---|
| ☒ | ☐ | ChIP-seq |
| ☒ | ☐ | Flow cytometry |
| ☒ | ☐ | MRI-based neuroimaging |

## Plants

| | |
|---|---|
| Seed stocks | *Report on the source of all seed stocks or other plant material used. If applicable, state the seed stock centre and catalogue number. If plant specimens were collected from the field, describe the collection location, date and sampling procedures.* |
| Novel plant genotypes | *Describe the methods by which all novel plant genotypes were produced. This includes those generated by transgenic approaches, gene editing, chemical/radiation-based mutagenesis and hybridization. For transgenic lines, describe the transformation method, the number of independent lines analyzed and the generation upon which experiments were performed. For gene-edited lines, describe the editor used, the endogenous sequence targeted for editing, the targeting guide RNA sequence (if applicable) and how the editor was applied.* |
| Authentication | *Describe any authentication procedures for each seed stock used or novel genotype generated. Describe any experiments used to assess the effect of a mutation and, where applicable, how potential secondary effects (e.g. second site T-DNA insertions, mosiacism, off-target gene editing) were examined.* |
